# Aortic Valve Annular Geometry in Athletes Practicing Sports with High Dynamics—A Detailed Three-Dimensional Speckle-Tracking Echocardiographic Investigation from the MAGYAR-Sport Study

**DOI:** 10.3390/biomedicines14051053

**Published:** 2026-05-06

**Authors:** Attila Nemes, Nóra Ambrus, Csaba Lengyel

**Affiliations:** Department of Medicine, Albert Szent-Györgyi Medical School, University of Szeged, H-6725 Szeged, Hungary; ambrusnora@gmail.com (N.A.); lengyel.csaba@med.u-szeged.hu (C.L.)

**Keywords:** left-ventricular strain, speckle-tracking echocardiography, sport, aortic valve annulus, three-dimensional

## Abstract

**Background**. Physiological remodeling resulting from chronic exercise-induced volume and pressure overload is a well-recognized characteristic of the athlete’s heart. This study aimed to explore potential changes in three-dimensional speckle-tracking echocardiography-derived aortic valve annular (AVA) dimensions and dynamics in elite athletes engaged in high-dynamic sports with varying degrees of static components. Furthermore, we sought to determine whether these parameters differ depending on the magnitude of the sport’s dynamic component. **Methods.** The athlete cohort included 56 individuals and was divided into three groups based on the static component of their sport: C. I. (high dynamic/low static) consisted of 13 elite athletes (mean age: 22.7 ± 3.8 years, 5 males), C. II. (high dynamic/moderate static) consisted of 18 elite athletes (mean age: 23.0 ± 4.6 years, 6 males) and C. III. (high dynamic/high static) consisted of 25 elite athletes (mean age: 21.7 ± 4.5 years, 9 males). Data of athletes were compared with those of 38 age- and sex-matched healthy non-athletic individuals (mean age: 23.8 ± 2.5 years, 14 males). **Results**. AVA dimensions did not differ significantly between athletes and controls nor among the athlete subgroups. AVA plane systolic excursion (AAPSE) was increased in all athletes compared with controls (1.31 ± 0.30 cm vs. 1.18 ± 0.36 cm, *p* < 0.05). All athletes showed a significantly larger proportion of individuals showing larger end-diastolic AVA than end-systolic AVA (55% vs. 24%, *p* < 0.05). All athletes demonstrated reduced basal LV-RS (26.5 ± 13.9% vs. 31.5 ± 13.2%, *p* < 0.05) and increased basal LV-LS (−21.4 ± 4.4 vs. 19.9 ± 4.2%, *p* < 0.05) compared with controls. This pattern of findings was consistent across all athlete subgroups. **Conclusions**. Although AVA is not dilated in elite athletes practicing dynamic sports, its spatial displacement, as represented by AAPSE, together with increased basal LV-LS and a higher proportion of larger end-diastolic AVA, is augmented, while basal LV-RS is reduced. These findings suggest a functional shift from radial contraction toward enhanced longitudinal dynamics. All these findings appear to be independent of the static component of dynamic sports.

## 1. Introduction

Physiological remodeling resulting from chronic exercise-induced volume and pressure overload is a well-recognized characteristic of the athlete’s heart. Physical exertion exposes the aortic valve (AV) to hemodynamic stress; however, it remains unclear whether athletes consistently exhibit larger AV dimensions [1,2]. More nuanced investigations have demonstrated heterogeneous changes in elite athletes, depending on the type and intensity of training [3,4]. Advanced cardiovascular imaging modalities, such as three-dimensional (3D) speckle-tracking echocardiography (STE), provide robust frameworks for comprehensive cardiovascular assessment [5,6,7,8,9]. Although the AV plays a crucial role in central hemodynamics [1,2], potential alterations in aortic valve annular (AVA) dimensions and dynamics in athletes remain largely unexplored. Modern 3DSTE is suitable not only for the accurate assessment of the sphincter-like motion of the valvular annuli (like AVA) throughout the cardiac cycle but also for quantifying their spatial displacement, such as the AVA plane systolic excursion (AAPSE), which was previously measured primarily via M-mode echocardiography [10,11,12]. Since the AV and the surrounding left ventricle (LV) form an integral unit—with spatial motion and function of the AVA largely depending on the surrounding LV tissue—their combined assessment is of essential importance [3,12]. As supported by the previous literature, LV strains representing LV contractility are enhanced in elite athletes practicing sports with high dynamics [13] and can be obtained via 3DSTE simultaneously with AVA and AAPSE measurements [12,14]. Hypothetically, questions arise as to whether significant changes occur in AVA dimensions and as to the extent of AAPSE as a consequence of elite sports and whether these show associations with regional LV function. Accordingly, the present study aimed to use 3DSTE to determine potential changes in AVA dimensions and dynamics in elite athletes engaged in high-dynamic sports with varying degrees of static components. Furthermore, we sought to investigate whether these parameters are associated with basal regional LV function, as assessed by myocardial strain analysis, and whether differences exist according to the magnitude of the sport’s dynamic component. To the best of the authors’ knowledge, no similar study has been conducted to date. The study was conducted in accordance with the STROBE reporting checklist.

## 2. Patients and Methods

**Participants.** This retrospective cohort study was conducted at the Department of Medicine, University of Szeged. Any registered elite athlete engaged in any competitive sport was eligible to participate in the study on a voluntary basis. Athlete recruitment and the examinations were conducted between 2014 and 2017. The athlete cohort included 56 individuals (mean age: 22.3 ± 4.5 years, 20 males) representing 8 different sports. Athletes were categorized according to the modified Mitchell classification of the American College of Cardiology based on the static and dynamic components of their training [4]. Individual competitive athletics are classified based on two fundamental movement modalities: dynamic (A-B-C) and static (I-II-III). Every discipline is ranked by the intensity—low (A or I.), moderate (B or II.), or high (C or III.)—of the physical strain typically experienced during competition. Furthermore, this classification accounts for sports involving substantial impact hazards, such as forceful contact between participants or collisions with equipment and the terrain. It also evaluates the potential danger to the athlete or bystanders in the event of sudden loss of consciousness (syncope). The rise in the dynamic component is measured by the estimated percentage of maximal oxygen (max O_2_) uptake (A: low, <40% max O_2_; B: moderate, 40–70% max O_2_; C: high, 70% max O_2_) achieved, leading to an elevated cardiac output. Conversely, the growth of the static component is linked to the estimated percentage of maximal voluntary contraction (MVC) reached (I.: low, <20% MVC; II.: moderate, 20–50% MVC; III.: high, >50% MVC), which places an increasing pressure load on the cardiovascular system [4]. All participants were registered members of sports clubs and underwent regular medical surveillance. Exclusion criteria included cardiovascular risk factors, known diseases, and any pathological conditions. None of the subjects were receiving pharmacological treatment, and all 12-lead electrocardiographic (ECG) and laboratory results were within normal ranges. On average, the athletes had completed 8.7 ± 4.7 years of regular training prior to enrollment. Participation was voluntary, and all athletes who provided informed consent were included in the analysis. Based on the aforementioned classification, the elite athletes were divided into the following groups:

- Group 1 (C. I., high dynamic/low static) consisted of 13 elite athletes, including 12 football players and 1 orienteer (mean age: 22.7 ± 3.8 years, 5 males),
- Group 2 (C. II., high dynamic/moderate static) consisted of 18 elite athletes, including 11 runners, 4 basketball players and 3 handball players (mean age: 23.0 ± 4.6 years, 6 males),
- Group 3 (C. III., high dynamic/high static) consisted of 25 elite athletes, including 14 paddlers, 7 triathletes and 4 boxers (mean age: 21.7 ± 4.5 years, 9 males).

Data of athletes were compared with those of 38 age- and sex-matched healthy non-athletic individuals (mean age: 23.8 ± 2.5 years, 14 males) selected from a larger cohort of over 300 healthy subjects recruited between 2011 and 2017. Participants underwent comprehensive cardiovascular evaluation, including physical examination, laboratory testing, standard 12-lead ECG, and two-dimensional (2D) Doppler echocardiography, followed by 3DSTE data acquisition and analysis. All clinical findings in this group were within normal reference ranges. Exclusion criteria included smoking, regular medication use, obesity (body mass index ≥ 30 kg/m^2^), pregnancy, professional sports activity, and regular yoga practice. Furthermore, none of the subjects had a history of chronic disease. Athletes and matched controls were all caucasian Hungarians. This research is part of the ‘**M**otion **A**nalysis of the heart and **G**reat vessels b**Y** three-dimension**A**l speckle-t**R**acking echocardiography in **Sports**men **(MAGYAR-Sport) Study**’, the purpose of which was to investigate elite sport-related changes in myocardial mechanics by 3DSTE (‘magyar’ means ‘Hungarian’ in the Hungarian language) [13]. The investigation was conducted in adherence with the Declaration of Helsinki (2013 revision) [15] and was approved by the Institutional and Regional Human Biomedical Research Committee at the University of Szeged, Hungary (registration no. 71/2011, extended on 17 March 2025). All participants provided written informed consent prior to their inclusion in the study.

**Two-dimensional Doppler echocardiography.** Echocardiographic examinations were performed using a Toshiba Artida^®^ system (Toshiba Medical Systems, Tokyo, Japan) equipped with a 1–5 MHz PST-30BT phased-array transducer. Routine assessments included cardiac chamber dimensions and LV ejection fraction using the Simpson method, in accordance with current guidelines, and Doppler assessment included evaluation of valvular regurgitation and stenosis, as well as transmitral early (E) and late (A) diastolic inflow velocities and their ratio to characterize LV diastolic function [16]. All examinations, including 3DSTE, were conducted by a single expert (AN). Analyses were performed in triplicate, and the resulting mean values were used for the study.

**Three-dimensional speckle-tracking echocardiography.** 3DSTE was performed by the same cardiac ultrasound tool after switching to a dedicated 2.5 MHz PST-25SX matrix phased-array transducer (Toshiba Medical Systems, Tokyo, Japan). First, 3D echocardiographic datasets were obtained from the apical window after optimizing image quality (gain, magnitude, etc.). To ensure optimal quality, six sub-volumes were acquired over six consecutive cardiac cycles during a single breath-hold, maintaining stable R-R intervals on the ECG. The software then automatically merged the sub-volumes into a full 3D dataset. Offline analysis was performed using the 3D Wall Motion Tracking software (Toshiba Medical Systems, Tokyo, Japan) [5,6,7,8,9].

**Figure 1:**
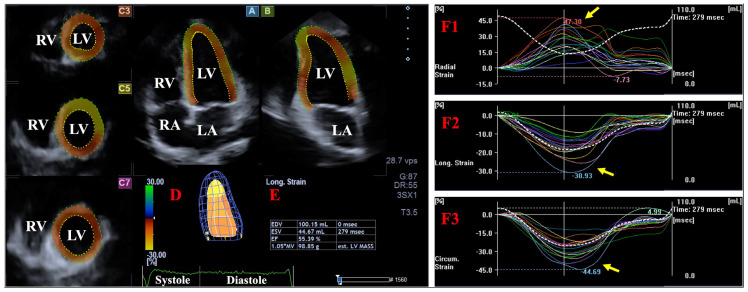
Visual representation of the imaging planes employed in the evaluation: apical four-chamber (**A**) and two-chamber (**B**) long-axis views, along with short-axis cross-sections at the apical (**C3**), midventricular (**C5**), and basal (**C7**) regions of the left ventricle (LV). Panel (**D**) shows the virtual three-dimensional reconstruction of the LV generated by the software, while the related volumetric parameters are displayed in Panel (**E**). Panels (**F1**–**F3**) present the global (white) and segmental (colored) time-strain curves for radial, longitudinal, and circumferential LV deformation, respectively, alongside the time–LV volume change curve (dashed white curve). Yellow arrow represents maximum of the LV strains at end-systole. Abbreviations: EDV: end-diastolic volume; EF: ejection fraction; ESV: end-systolic volume; RA: right atrium; RV: right ventricle; LA: left atrium; LV: left ventricle.

To assess LV strains, three cross-sectional planes were reconstructed automatically from apical four-chamber (AP4CH) and two-chamber (AP2CH) long-axis views. The investigator marked the apical LV endocardial border as well as the septal and lateral edges of the LV and the mitral annulus. A 3D virtual LV model was then created following sequential processing and automated boundary detection. Peak unidirectional end-systolic LV strains—radial (RS), circumferential (CS), and longitudinal (LS)—were measured at the basal level. These functional parameters represent myocardial LV thickening/thinning, circumferential narrowing/widening, and longitudinal shortening/lengthening, respectively [13,17] (Figure 1).

**Figure 2:**
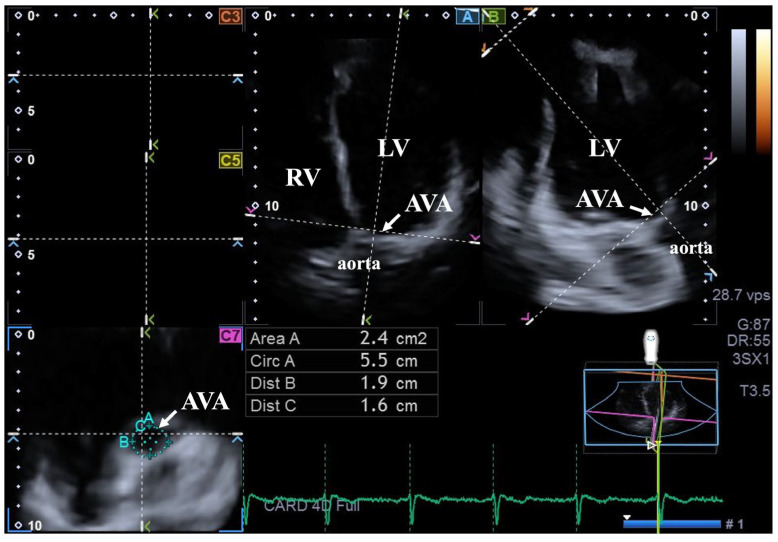
Assessment of aortic valve annular dimensions using three-dimensional speckle-tracking echocardiography. (**A**) Apical four-chamber and (**B**) apical two-chamber long-axis views, alongside a (**C**) cross-sectional view of the aortic valve annulus optimized from images (**A**) and (**B**). The aortic valve annular plane is indicated by white arrows. Abbreviations: LV: left ventricle; AVA: aortic valve annulus; Area: AVA area; Circ: AVA perimeter; Dist B: maximum AVA diameter; Dist C: minimum AVA diameter.

For AVA assessment, optimal LV longitudinal planes were defined using AP4CH and AP2CH long-axis views. Following visualization of the aorta and the aortic valve, imaging settings were optimized, and planes were aligned parallel to the aortic root centerline. A perpendicular C7 cross-sectional view was used for AVA measurements, ensuring exclusion of the LV outflow tract and the sinuses of Valsalva. At both end-diastole and end-systole, the following AVA parameters were measured using planimetry: minimum and maximum diameters (AVA-Dmin, AVA-Dmax) as well as area (AVA-A) and perimeter (AVA-P). AVA plane systolic excursion (AAPSE) was also measured, reflecting the spatial displacement of the AVA plane throughout the cardiac cycle [12,14,18]. All LV and AVA measurements were averaged over 5 cardiac cycles. Beyond averaging, inter-cycle variability was minimized by using ECG-gated assessments, ensuring that all measurements were performed at the same point in the cardiac cycle (all subjects were in sinus rhythm). Moreover, all measurements were blinded (Figure 2).

**Statistical analysis.** Data were presented as mean ± standard deviation (SD) or as counts and percentages (*n* [%]), as appropriate. Normality and homogeneity of variances were assessed using Levene’s test. Group comparisons were performed using independent samples *t*-tests for continuous variables and Fisher’s exact test for categorical variables. Pearson’s correlation coefficients were calculated for correlation analyses. Reproducibility of 3DSTE-derived AVA parameters was assessed using inter- and intra-observer variability in 30 healthy individuals, expressed as mean difference ± 2SD and further validated by intraclass correlation coefficients (ICCs). To account for multiple comparisons, one-way analysis of variance (ANOVA) followed by Bonferroni’s correction was applied, where appropriate. A post hoc power analysis was conducted using the G*Power software (version 3.1) based on the observed effect size, an alpha level of 0.05, and the actual sample size. A *p*-value of less than 0.05 was considered statistically significant. Statistical analyses were performed using IBM SPSS Statistics for Windows, version 29.0 (IBM Corp., Armonk, NY, USA).

## 3. Results

**Clinical and two-dimensional Doppler echocardiographic data.** The duration of elite sports participation was 11.8 ± 3.6 years in Group 1, 9.2 ± 5.5 years in Group 2, and 7.0 ± 4.2 years in Group 3. No significant differences were observed in the proportion of athletes in their active competitive season across the three groups (*n* = 4 [31%], *n* = 8 [44%], and *n* = 8 [32%], respectively; Table 1). Conventional echocardiographic data for the left atrium and LV are also summarized in Table 1. Height (175.9 ± 9.5 cm, 176.6 ± 8.3 cm, 181.2 ± 7.7 cm, 174.1 ± 10.1 and 176.0 ± 7.0 cm, respectively), body weight (70.6 ± 10.7 kg, 70.8 ± 10.9 kg, 72.1 ± 8.3 kg, 68.3 ± 14.7 kg, 71.9 ± 10.4 kg, respectively) and body mass index (22.1 ± 2.3 kg/m^2^, 22.6 ± 2.6 kg/m^2^, 21.9 ± 2.0 kg/m^2^, 22.4 ± 3.1 kg/m^2^ and 23.1 ± 2.5 kg/m^2^, respectively) did not differ significantly between controls, the overall athletes cohort, or among CI, CII and CIII subgroups.

**Two-dimensional Doppler echocardiography.** No significant differences were observed in routine 2D echocardiographic parameters between controls and athletes or among the C. I., C. II. and C. III. subgroups. However, interventricular septal thickness was significantly increased in C. III. subgroup compared with controls.

**Three-dimensional speckle-tracking echocardiography.** AVA dimensions did not differ significantly between athletes and controls nor among the athlete subgroups. AAPSE was increased in all athletes compared with controls. In addition, a significantly higher proportion of athletes exhibited larger end-diastolic AVA than end-systolic AVA. Athletes demonstrated reduced basal LV-RS and increased basal LV-LS compared with controls. This pattern was consistent across all athlete subgroups (Table 2).

**Correlations and post hoc power analysis.** AAPSE did not show significant correlations with basal LV-RS (r = −0.03, *p* = 0.84), LV-CS (r = −0.02, *p* = 0.89), or LV-LS (r = −0.16, *p* = 0.35) in controls nor in elite athletes (r = −0.11, *p* = 0.47; r = −0.26, *p* = 0.09 and r = 0.03, *p* = 0.85, respectively). A post hoc power analysis was conducted to assess the robustness of the observed differences. Based on the current sample size, the analysis yielded a large effect size (Cohen’s d = 0.8) and a statistical power of 0.68 at an alpha level of 0.05.

**Reproducibility of 3DSTE-derived AVA assessments.** Table 3 summarizes the intra-observer (repeated measurements by the same investigator) and inter-observer (evaluations by two independent researchers) variability in end-systolic and end-diastolic AVA parameters, including minimum and maximum perimeters, areas, and diameters. Results are expressed as mean difference ± 2SD and supported by ICCs.

## 4. Discussion

Professional sports are associated with a complex pattern of cardiac remodeling, encompassing both volumetric and functional alterations in the cardiac chambers and dilation of the valvular annuli, commonly referred to as the “athlete’s heart” [1,2]. According to the Mitchell classification, different sports impose varying static and dynamic loads, which differentially influence cardiac remodeling and therefore require precise quantification [4].

The AV functions as the gateway to the LV, with its cyclic opening and closing regulating stroke volume into the systemic circulation [19]. While conventional imaging studies have suggested that athletic activity may result in a small but significantly larger aortic root diameter at the AVA, this difference was minor and clinically insignificant [1]. In contrast to other cardiac structures, the aortic root appears to exhibit limited structural remodeling in response to exercise, with dynamic training inducing only minimal expansion and static training showing negligible effects [2].

State-of-the-art 3DSTE offers a superior diagnostic approach, enabling simultaneous measurement of LV strains —providing global and regional quantitative indices of LV contractility—and AVA dimensions throughout the cardiac cycle from a single, digitally acquired 3D echocardiographic dataset [12,13,14]. Moreover, this technique allows for the quantification of AVA displacement at the same time [12,14]. The validity of 3D echocardiographic measurements has been well established [20,21,22], with available reference values from large cohorts such as the MAGYAR-Healthy Study [18,23]. This approach facilitates a more profound assessment of (patho)physiological mechanisms in elite athletes, which have hitherto remained unexplored. Although enhanced regional LV function is a well-known phenomenon in elite athleticism [13], previous research failed to capture the area, perimeter, and systolic–diastolic variations in the AVA, to determine AAPSE, or to examine the correlation between AVA dimensions, AAPSE and LV deformation in this specific population.

The present study offers several clinical and physiological insights. First, it confirms that 3DSTE can simultaneously derive global and regional LV strains and measure AVA dimensions at end-diastole and end-systole, along with AAPSE. Second, AVA dimensions are not dilated in elite athletes practicing high-dynamic sports compared with matched controls. Third, higher AAPSE values together with enhanced basal LV-LS in athletes suggest increased spatial displacement of the AVA. However, basal LV-RS was reduced, suggesting a form of functional reorganization, whereby the LV adapts less through radial ‘squeezing’ and more through ‘stretching and shortening’ [13]. Fourth, while the general population typically exhibits larger AVA dimensions in end-systole in approximately two-thirds of cases [24], our results suggest that this ratio is effectively reversed in elite athletes practicing dynamic sports, which can be considered as a sign of functional remodeling as well. Finally, these adaptive alterations showed no significant associations with the specific magnitude of the sport’s dynamic component, a finding which is consistent with previous results [4].

Several questions remain unanswered. Firstly, it is unclear to what extent these findings are reversible and what occurs when an athlete temporarily or permanently ceases sports activity [25,26]. Furthermore, whether similar adaptations occur in athletes engaged predominantly in static sports warrants further clinical studies. The question may also arise as to the extent to which the results detailed above are suitable for screening athletes and whether these findings possess diagnostic and prognostic significance. Furthermore, it remains to be determined whether the presented results represent physiological adaptations associated with elite sports or, potentially, early asymptomatic pathological signs of remodeling. Addressing these questions requires further investigations.

## 5. Limitation Section

- Conventional 2D echocardiography still provides superior image quality compared with 3DSTE, primarily due to its higher temporal and spatial resolution. Furthermore, the larger physical dimensions of the 3DSTE transducer make it more challenging to achieve an optimal acoustic window. In addition, the data acquisition process, which requires merging six sub-volumes over six consecutive cardiac cycles to enhance resolution, is inherently susceptible to motion artifacts and stitching inaccuracies [5,6,7,8,9].
- Regarding further technical limitations, valvular regurgitation was evaluated using visual and qualitative evaluation only; more advanced quantitative grading methods were not applied in the present study.
- Although alternative imaging modalities (including cardiac computer tomography, magnetic resonance imaging, or other ultrasound modalities) may provide more detailed insights into AVA morphology, such comprehensive evaluations were beyond the scope of the present study.
- The total sample size was relatively small, and its further division into three subgroups limited the possibility of gender-specific analyses. Such analyses would have enhanced the clinical relevance and scientific robustness of the study. However, the gender distribution across the studied groups was similar.
- The compared groups were not fully homogeneous; a comparison of athletes within the same sporting disciplines across groups would have been preferable.
- A key limitation of this research is the absence of a formal a priori power analysis. Although the study involved 56 participants, the subgroup analyses (Table 1 and Table 2) were based on smaller cohorts (*n* ≤ 25). This may have reduced the statistical power to identify subtle associations, increasing the risk of Type II errors. While the baseline characteristics remained relatively homogeneous, these results should be viewed as exploratory. Future studies with larger, pre-determined sample sizes are necessary to validate these findings.

## 6. Conclusions

Although AVA is not dilated in elite athletes practicing dynamic sports, its spatial displacement represented by AAPSE, together with increased basal LV-LS and a higher proportion of larger end-diastolic AVA, is augmented, while basal LV-RS is reduced. These findings suggest a functional shift from radial contraction toward enhanced longitudinal dynamics. All these findings appear to be independent of the static component of dynamic sports.

## Figures and Tables

**Table 1:** Two-dimensional echocardiographic data of athletes and matched controls.

	Controls (*n* = 38)	All Athletes (*n* = 56)	Group 1 (*n* = 13) (High Dynamic/ Low Static)	Group 2 (*n* = 18) (High Dynamic/Moderate Static)	Group 3 (*n* = 25) (High Dynamic/High Static)
**LA-D (mm)**	37.8 ± 3.0	36.4 ± 3.2	35.1 ± 3.4	36.3 ± 2.4	37.2 ± 3.5
**LV-ED-D (mm)**	48.6 ± 3.6	49.6 ± 3.1	50.4 ± 3.4	48.4 ± 2.5	50.2 ± 3.1
**LV-ED-V (mL)**	107.2 ± 24.0	117.1 ± 20.5	120.8 ± 20.0	111.8 ± 12.7	119.5 ± 24.5
**LV-ES-D (mm)**	32.2 ± 3.2	30.4 ± 2.6	31.6 ± 2.4	29.1 ± 1.9	30.8 ± 2.6
**LV-ES-V (mL)**	37.4 ± 8.3	37.5 ± 8.5	41.3 ± 9.1	33.7 ± 6.3	38.7 ± 8.5
**IVS (mm)**	8.9 ± 1.0	9.4 ± 1.3	9.4 ± 1.4	8.9 ± 1.1	9.7 ± 1.2 *
**LV-PW (mm)**	9.1 ± 1.2	9.2 ± 1.2	9.2 ± 1.3	8.8 ± 1.1	9.6 ± 1.2
**LV-EF (%)**	65.0 ± 4.1	68.4 ± 3.9	66.0 ± 3.4	70.0 ± 3.9	68.3 ± 3.5
**E (cm/s)**	87.3 ± 14.8	94.7 ± 15.7	84.4 ± 13.9	96.3 ± 14.7	98.2 ± 15.2
**A (cm/s)**	57.0 ± 10.3	61.0 ± 10.2	60.4 ± 11.1	61.4 ± 6.3	61.1 ± 11.9

* *p* < 0.05 vs. controls Abbreviations: A = late transmitral flow velocity, D = diameter, E = early transmitral flow velocity, ED = end-diastolic, EF = ejection fraction, ES = end-systolic, IVS = interventricular septum, LA = left atrium; LV = left ventricle; PW = posterior wall, V = volume.

**Table 2:** Aortic valve annular dimensions, systolic excursion of its plane and basal left-ventricular strains as assessed simultaneously by three-dimensional speckle-tracking echocardiography in athletes and matched controls.

	Controls (*n* = 38)	All Athletes (*n* = 56)	Group 1 (*n* = 13) (High Dynamic/ Low Static)	Group 2 (*n* = 18) (High Dynamic/Moderate Static)	Group 3 (*n* = 25) (High Dynamic/High Static)
**AVA-Dmax-D (cm)**	2.01 ± 0.36	2.04 ± 0.28	2.00 ± 0.32	2.04 ± 0.28	2.07 ± 0.26
**AVA-Dmin-D (cm)**	1.81 ± 0.33	1.78 ± 0.26	1.78 ± 0.26	1.79 ± 0.22	1.76 ± 0.28
**AVA-A-D (cm^2^)**	3.07 ± 0.98	3.32 ± 0.75	3.38 ± 0.61	3.23 ± 0.95	3.34 ± 0.64
**AVA-P-D (cm)**	6.23 ± 0.99	6.41 ± 0.77	6.42 ± 0.74	6.38 ± 0.89	6.43 ± 0.70
**AVA-Dmax-S (cm)**	2.06 ± 0.34	1.95 ± 0.28	1.95 ± 0.22	1.91 ± 0.32	1.98 ± 0.27
**AVA-Dmin-S (cm)**	1.83 ± 0.26	1.71 ± 0.27	1.69 ± 0.23	1.69 ± 0.29	1.72 ± 0.28
**AVA-A-S (cm^2^)**	3.29 ± 0.93	3.09 ± 0.81	3.08 ± 0.53	2.92 ± 0.92	3.23 ± 0.82
**AVA-P-S (cm)**	6.46 ± 0.89	6.24 ± 0.79	6.28 ± 0.54	6.03 ± 0.93	6.36 ± 0.76
**AAPSE (cm)**	1.18 ± 0.36	1.31 ± 0.30 *	1.35 ± 026	1.25 ± 0.33	1.33 ± 0.28
**larger AVA-A-D/AVA-A-S**	9 (24)	31 (55)*	7 (54) *	11 (61) *	13 (52) *
**equal AVA-A-D/AVA-A-S**	3 (8)	11 (20)	3 (23)	4 (22)	4 (16)
**smaller AVA-A-D/AVA-A-S**	26 (68)	14 (25) *	3 (23) *	3 (17) *	8 (32) *
**RS_basal_ (%)**	31.5 ± 13.2	26.5 ± 13.9 *	23.6 ± 8.6	27.7 ± 11.3	26.5 ± 16.2
**CS_basal_ %)**	−26.2 ± 4.7	−25.0 ± 5.5	−23.6 ± 5.3	−26.0 ± 5.5	−25.0 ± 6.0
**LS_basal_ (%)**	−19.9 ± 4.2	−21.4 ± 4.4 *	−21.7 ± 4.7	−21.5 ± 3.9	−21.1 ± 4.7

* *p* < 0.05 vs. controls **Abbreviations:** AVA = aortic valve annulus; Dmax = maximum diameter; Dmin = minimum diameter; A = area; P = perimeter; D = end-diastolic; S = end-systolic; AAPSE = aortic valve annular plane systolic excursion; RS = radial strain; CS = circumferential strain; LS = longitudinal strain.

**Table 3:** Intra- and interobserver variability for three-dimensional speckle-tracking echocardiography-derived assessment of aortic valve annular dimensions and aortic valve plane systolic excursion.

	Intra-Observer Agreement		Inter-Observer Agreement	
	Mean ± 2SD Difference in Values Obtained by 2 Measurements of the Same Observer	Correlation Coefficient Between Measurements of the Same Observer	Mean ± 2SD Difference in Values Obtained by 2 Observers	Correlation Coefficient Between Independent Measurements of 2 Observers
**AAPSE (cm)**	−0.03 ± 0.12	0.92 (*p* < 0.01)	−0.04 ± 0.15	0.90 (*p* < 0.01)
**AVA-Dmax-D (cm)**	−0.03 ± 0.21	0.90 (*p* < 0.01)	−0.05 ± 0.16	0.88 (*p* < 0.01)
**AVA-Dmin-D (cm)**	−0.03 ± 0.18	0.88 (*p* < 0.01)	−0.05 ± 0.20	0.91 (*p* < 0.01)
**AVA-A-D (cm^2^)**	−0.11 ± 0.56	0.91 (*p* < 0.01)	−0.11 ± 0.46	0.92 (*p* < 0.01)
**AVA-P-D (cm)**	−0.03 ± 0.61	0.91 (*p* < 0.01)	−0.12 ± 0.45	0.93 (*p* < 0.01)
**AVA-Dmax-S (cm)**	0.03 ± 0.32	0.93 (*p* < 0.01)	0.05 ± 0.32	0.95 (*p* < 0.01)
**AVA-Dmin-S (cm)**	0.04 ± 0.29	0.85 (*p* < 0.01)	0.02 ± 0.35	0.84 (*p* < 0.01)
**AVA-A-S (cm^2^)**	0.11 ± 0.65	0.91 (*p* < 0.01)	0.10 ± 0.71	0.93 (*p* < 0.01)
**AVA-P-S (cm)**	−0.03 ± 0.57	0.92 (*p* < 0.01)	0.02 ± 0.56	0.93 (*p* < 0.01)

Abbreviations: AVA = aortic valve annulus, Dmax = maximum diameter, Dmin = minimum diameter, A = area, P = perimeter, D = end-diastolic, S = end-systolic, AAPSE = aortic valve plane systolic excursion.

## Data Availability

The data presented in this study are available on request from the corresponding author.
